# Novel Insights Into the Role of Glycans in the Pathophysiology of Glomerular Endotheliosis in Preeclampsia

**DOI:** 10.3389/fphys.2018.01470

**Published:** 2018-10-23

**Authors:** M. F. Galvis-Ramírez, J. C. Quintana-Castillo, J. C. Bueno-Sanchez

**Affiliations:** ^1^Grupo Reproducción, Sede de Investigación Universitaria, Universidad de Antioquia, Medellín, Colombia; ^2^Grupo Infettare, Facultad de Medicina, Universidad Cooperativa de Colombia, Medellín, Colombia

**Keywords:** glomerular endothelia dysfunction, preeclampsia, heparan sulfate (HS), systemic inflammatory response, renal damage

## Abstract

The polysaccharide heparan sulfate is ubiquitously expressed as a proteoglycan in extracellular matrices and on cell surfaces. In the glomerular filtration barrier, the action of the heparan sulfate is directly related to the function of glomerular filtration, mostly attributed to the sulfated domains that occur along the polysaccharide chain, as evidenced by fact that release of fragments of heparan sulfate by heparanase significantly increases the permeability of albumin passage through the glomerular endothelium, event that originates proteinuria. This review aims to show the importance of the structural domains of heparan sulfate in the process of selective permeability and to demonstrate how these domains may be altered during the glomerular inflammation processes that occur in preeclampsia.

## Introduction

Preeclampsia (PE) is characterized as a progressive multisystemic disorder diagnosed by hypertension and proteinuria after 20 weeks of pregnancy. In some clinical cases, proteinuria is absent, and the disease is diagnosed by another clinical findings such as thrombocytopenia, elevated liver enzymes, among others (Visintin et al., 2010; ACOG, 2013). Preeclampsia is accompanied by a mild to severe microangiopathy of target organs, such as the placenta, kidney, liver, and brain (Gilstrap and Ramin, 2002). The placental tissue is a determinant in the establishment of the disease, which is always “cured" after the delivery of the placenta. Evidence related to the role of the placenta in this disease has led to the proposal that failed trophoblast invasion is the central pathogenic mechanism (Kang et al., 2011). Thus, some authors have suggested that preeclampsia is a disease of two stages: in the first stage, poor placentation and the subsequent hypoxic and oxidative damage of trophoblast occur; whereas in the second stage, an endothelial dysfunction and hypertensive signs are evidenced (Goldman-Wohl and Yagel, 2009; Kang et al., 2011).

Preeclampsia exhibits diverse clinical manifestations such as mild or severe, early onset (<34 weeks) or late onset (>34 weeks), or the presence or absence of intrauterine growth retardation (Gilstrap and Ramin, 2002; Giachini et al., 2017). Accumulating evidence has shown that pathological features of preeclampsia involve a shallow trophoblast invasion and poor spiral artery remodeling, resulting in placental hypoperfusion. These events occur in the first trimester of pregnancy and initially compromise maternal–fetal interface one, but they result in increased production of antiangiogenic and inflammatory factors (Schiessl, 2007; Palei et al., 2013). Additionally, hypertensive disorders of pregnancy are associated with endothelial damage of target organs including the kidneys. Although the clinical manifestations of PE are present after 20 weeks of pregnancy, some evidences indicate that the pathophysiological changes leading to the disease occur in the placental bed during the placentation process. Reported findings suggest that these events promote exacerbated systemic inflammatory responses and the subsequent production of soluble factors, including proinflammatory cytokines, as well as changes in the ratio of angiogenic/proangiogenic factors, which are the determinant variables of changes in the endothelium, the target organ of PE (Levine et al., 2004; Goswami et al., 2006).

Pregnancy has been described as a controlled state of mild inflammation, whereas a state of exacerbated inflammation apparently occurs in PE (Sargent et al., 2007). Our research group has shown that the activation of natural killer (NK) cells is increased compared with other cell populations, which is evidenced by the higher production of both pro- and anti-inflammatory cytokines in patients with early-onset severe PE (Bueno-Sanchez et al., 2013). However, other authors have found that the intracellular production of pro-inflammatory cytokines [interleukin-1 beta (IL-1β), tumor necrosis factor-alpha (TNF-α), IL-6, and IL-8] by circulating maternal monocytes is increased in patients with PE (Luppi and Deloia, 2006). The increased production of proinflammatory cytokines from different cellular sources (NK cells according to our data and monocytes or neutrophils according to other studies) initially triggers inflammatory changes in the vascular endothelium and then compromises the endothelium of other organs (Stella and Sibai, 2006). Accordingly, Rops et al. (2008) have shown that IL-1β and TNF-α may alter the expression of glycocalyx structures, including the specific domains of heparan sulfate (HS), a glycosaminoglycan present in the renal glomerular endothelium.

Histopathological changes in the glomerular endothelium, which partly explain the proteinuria observed in women with PE, are among the aforementioned changes (Karumanchi et al., 2005). Studies have shown that the structural integrity of the glomerular filtration barrier (GFB) consists of two cellular components, the glomerular endothelium, and podocytes, as well as an extracellular matrix structure with a high content of glycoconjugates, including the glomerular basement membrane (Karumanchi et al., 2005). Reports have suggested that the structural integrity of the GFB is crucial for proper functioning during the process of renal filtration. Thus, a structural damage of the glomerular filtration barrier can be explained by a competitive blocking of vascular endothelial growth factor (VEGF) in the murine model after administration of a synthetic VEGF antagonist. These mice presented nephrotic-range proteinuria and hypertension compared with controls (Eremina et al., 2008). These findings raise the possibility of assessing the biochemical aspects of the glomerular endothelium, including its function in filtration, as a means of clarifying the mechanisms of renal damage that occur in women with PE.

We have reported evidence of changes in the membrane distribution of podocin and CD2AP in podocytes, which are cellular components of the GFB, when stimulated with sera from women with PE (Henao et al., 2008). However, few studies have investigated the role of the glomerular endothelium in PE. In turn, Singh et al. (2007) have proposed a key role for the glycocalyx of glomerular endothelial cells in the GFB-mediated restriction of albumin permeability, suggesting elements that can be used to evaluate renal damage in PE. Among these carbohydrates, glycosaminoglycans, particularly HS glycosaminoglycans, are components of the glomerular endothelium glycocalyx that are associated with structures referred to as proteoglycans. HS activity is directly involved in glomerular filtration, and its involvement is mostly attributed to the sulfated domains throughout the polysaccharide chain. This is evidenced by the heparanase-mediated release of HS fragments, which significantly increase albumin permeability through the glomerular endothelium, albeit without affecting the transendothelial electrical resistance (Singh et al., 2007).

Based on the above evidence, this review aims to show the possible role of the structural domains of HS in the process of selective permeability through the GFB and how these domains may be altered during the glomerular inflammation processes that occur in PE.

## The Sulfated Domains of HS Mediate Its Interactions with Different Proteins

Heparan sulfate proteoglycans (HSPGs) are biomolecules with structural and regulatory functions that are capable of establishing ionic interactions with several proteins through structural regions in the polysaccharide chains. HSPGs consist of a protein core covalently bound to the side chains of HS (Coombe and Kett, 2005). This polysaccharide consists of alternating units of glucuronic acid (GlcA) and *N-*acetylglucosamine (GlcNAc), which undergo various enzymatic modifications including *N*-deacetylation and *N*-sulfation of the GlcNAc units (Carbon 6 and Carbon 3), epimerization in the C5 of GlcA to iduronic acid (IdoA) and *O*-sulfation in Carbon 2 (Kreuger et al., 2006). The various modifications do not occur randomly along the polysaccharide chain. Instead, they have the distribution of a typical domain because of limitations imposed by substrate specificity (and factors that are still unknown). Disaccharide units alternating between GlcNS and IdoA with 2-*O*-sulfate substitutions (NS domains) provide highly sulfated regions that may be interspersed with *N*-acetylated GlcNAc and GlcA regions lacking substitutions by sulfate group residues (NA domains). Similarly, forms with alternating NS/NA domains may also be found, albeit without *O*-sulfate groups (Maccarana et al., 1996; Sasisekharan and Venkataraman, 2000). The study of HS structures, which have been characterized in several mouse tissues, shows that the disaccharide composition, total degree of *N*- and *O*-sulfations and domain organization are specific to each tissue (Ledin et al., 2004). Furthermore, immunohistochemistry analyses have revealed the selective expression of different HS glycotopes in rat kidney tissues (van den Born et al., 1995; van Kuppevelt et al., 1998). The structural complexity of HS domains is even greater when considering that the HS chains may be modified after biosynthesis by endo-6-*O*-sulfatases, which generate HS fragments that have been functionally implicated in various signaling pathways (Ai et al., 2003). Furthermore, HS domains may undergo endoglycosidic cleavage by heparanases, thereby releasing extracellular HS fragments (Vlodavsky et al., 1999). In summary, these observations suggest that HS biosynthesis is highly regulated at different points of enzymatic processing in events that apparently vary with the tissue conditions or cellular environment.

This structural diversity could largely explain the interactions between HS and various proteins. Although the types of chemical interactions between HS and proteins have been difficult to elucidate, some proteins are known to require HS sequences with a well-defined length and structure, especially proteins whose net charge is positive, thereby suggesting that such interactions are not as nonspecific as initially thought.

The best characterized interaction between HS and a protein involves the binding of HS to antithrombin III, which is involved in hemostasis. Evidence first reported by Lindahl et al. (1980) shows that the pentasaccharide GlcNAc6S-GlcAGlcNS3S-IdoA-GlcNS is essential for the high-affinity interaction with antithrombin to occur and, therefore, for anticoagulant activity. However, these results most notably demonstrate the importance of specific HS structural domains, including the presence of a 3-sulfate group in the terminal glucosamine residue, which is crucial for such activity (Lindahl et al., 1980).

Another example of an interaction between HS and a protein involves a specific 3-*O*-sulfated domain of the GlcN3S unit that apparently mediates the specific binding of the herpes simplex virus (HSV) protein gD to the cell surface during viral infection (Shukla et al., 1999). According to some authors, in addition to this domain, the most common sulfate substituents in biosynthesis (3-*O* sulfate and 2-*N* sulfate) could also be specifically arranged in a sequence for selective binding to a protein (Salmivirta et al., 1996). Interaction studies involving the fibroblast growth factor protein family have shown that a particular type of sulfated domain (6-*O*-sulfate) could contribute more to the interaction than other domains (Salmivirta et al., 1996). These findings and the strict regulation of HS biosynthesis suggest that HS–protein interactions are mostly mediated by specific saccharide-binding domains with restricted specificity.

## The Glomerular Endothelial Glycocalyx Is a Barrier that Is Selectively Permeable to Albumin

Evidence shows that the NS domains of HS mediate its interactions with some proteins, and these interactions may be associated with its biological action. This suggests that the negative charges of the HS domains in the GFB could play a role in its selective permeability to positively charged proteins, including albumin, which are retained in the plasma. Conversely, small peptides, electrolytes, and even immunoglobulins are filtered into the urine during the glomerular filtration process (Singh et al., 2007).

**FIGURE 1:**
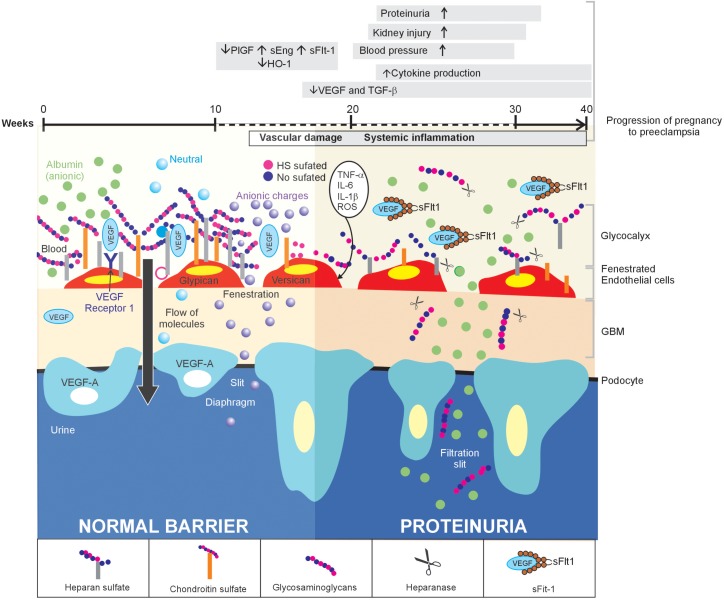
Inflammation of the glomerular endothelium causes the loss of HS, thereby triggering proteinuria in women with PE: a proposed model. In normal conditions (left side), the GFB performs its function of filtration, which is characterized by selective permeability to albumin due to HS in the glomerular endothelial glycocalyx, through its three layers. When a glomerular endothelial inflammation is established (right side), as is the case of PE, the increase in pro-inflammatory cytokines promotes the heparanase-mediated endoglucosidic activity of HS, which leads to the loss of anionic sites and, consequently, proteinuria.

The GFB is described as a set of three tissue layers: glomerular endothelium, glomerular basement membrane, and podocytes (Figure 1). Special large epithelial cells with cytoplasmic extensions (pedicels or foot processes) called podocytes are found toward the outer surface of the glomerular basement membrane and the fenestrated glomerular endothelium toward the innermost location, which is in contact with the plasma (Satchell, 2013). The GFB is highly selective and permeable to water and small molecules. However, for some proteins including albumin, only 0.008% of the plasma fraction is filtered into the urinary space compared with 0.2% through other systemic capillaries (Norden et al., 2001). Some authors have highlighted proteins expressed in the filtration diaphragm between pedicels because proteinuria is usually accompanied by changes in the distribution of these proteins, thereby suggesting a causal relationship between the expression of diaphragm proteins and albumin excretion. However, the size of the podocyte clefts is inconsistent with the size of albumin, which is much smaller, thereby raising questions of whether this could explain urinary albumin excretion as previously proposed (Haraldsson and Sorensson, 2004; Henao et al., 2008). Accordingly, some examples indicate that proteinuria may be found in diseases such as nephrotic syndrome even when podocyte integrity is maintained. Animal experiments show that the down-regulation of circulating VEGF or neutralization by either antibodies or Soluble fms-like tyrosine kinase-1 (sFIt-1) may play a key role in inducing proteinuria without changes in podocyte proteins in various renal diseases or PE, wherein a high level of sFIt-1 has been associated with the endothelial dysfunction observed in these women (Maynard et al., 2003; Sugimoto et al., 2003; Stillman and Karumanchi, 2007). This condition has also been examined in humans. A previous study involving 27 cases of nephrotic syndrome with minimal changes at different stages that assessed podocyte morphology using electron microscopy showed greater changes in podocyte pedicels in the more chronic forms of the disease. However, no significant differences in proteinuria were found between the study groups formed according to the time of evolution, thereby conclusively indicating that changes in podocytes are unrelated to protein loss (van den Berg et al., 2004).

By the other hand, it is known that HSPGs are abundant in the glomerular basal membrane and it has been thought to play a major role in the charge-selective glomerular filtration barrier. However, knockout of Ext3 (an enzyme that adds GlcNAc to the fixed tetrasaccharides on the core protein in proteoglycans) in podocytes and glomerular basal membrane did not lead to overt proteinuria (Aoki et al., 2018). Aoki et al. (2018) found a bumpy glomerular basal membrane and podocyte effacement by electron microscopy using a Extl3KO mice model compared with Diabetes Mellitus and high sodium intake mice groups, however they had some limitations in their study because the follow-up period was too short for remarkable renal impairments and establishment of hypertension. Additionally, renal function tests and HS urine measurements were not assessed in all groups of mice, which would help to support the possible role of HSPGs in the physiology of the GFB.

Thus, the summarized evidence shows that changes in podocytes or glomerular basal membrane fail to explain completely the onset of proteinuria, suggesting that the glomerular endothelium and its glycocalyx structures could be involved in selective permeability to albumin because this the first layer in contact with plasma and, therefore, the first structure to provide a barrier to albumin. Reported evidence on glomerular endothelium damage in diseases including PE, which is characterized by the presence of hypertension with the onset of proteinuria in most diagnosed cases, suggests that the endothelium may play a key role in the onset of proteinuria in this disease (Maynard et al., 2003). Other studies support a selective permeability layer (glycocalyx) at the endothelial level (Sorensson et al., 1998; Ohlson et al., 2000; Ciarimboli et al., 2003; Jeansson and Haraldsson, 2006).

The glomerular endothelium has two key structural characteristics. The first is a set of fenestrations or pores that are sufficiently large enough (36 Å or 3.6 nm) for water and other molecules, including albumin, to move through the endothelial cell layer (Levick and Smaje, 1987). This characteristic alone contradicts the aforementioned evidence implying the additional presence of a barrier within the fenestrations. The second component is the glycocalyx, which covers the fenestrations and has molecular and charge characteristics that could restrict albumin movement through the glomerular endothelium. Its complex structure consists of proteoglycans, including syndecan, glypican, or biglycan – with HS, *N-*, and *O* – glycoproteins containing sialic acid that is expressed on the endothelium luminal surface (Reitsma et al., 2007; Weinbaum et al., 2007). Jeansson was one of the first researchers to study in detail the selective permeability of the GFB in mice and proposed that hyaluronic acid, chondroitin sulfate, sialic acid, and HS are important for selective permeability because of their electrical charges (Jeansson and Haraldsson, 2006). A key observation is the increased ratio of HS and hyaluronic acid over sialic acid (Avasthi and Koshy, 1988). This observation in particular suggests that HS may make a greater contribution to the electrical charge of the GFB because hyaluronic acid lacks significant negative charges when compared with the sulfated domains of HS. This hypothesis may be supported by studies conducted by Singh et al. (2007), who assessed the glycocalyx structure in a glomerular endothelial cell line to examine its relevance to endothelial-selective permeability. They found that the removal of HS after treatment with human heparanase (the only endogenous enzyme described in mammals that degrades HS) was associated with increased albumin movement through the fenestrations without changing the transendothelial electrical resistance. These results suggest a possible role for the glycocalyx in restricting protein movement through the GFB and raise the possibility that heparanase levels in humans are related to proteinuria in kidney damage (Jin and Zhou, 2017). Additionally, the HS fragments induced by heparanase from GFB could contribute to release local proinflammatory cytokines or chemokines in the extracellular space, modifying the inflammatory response, and the endothelial glycocalix (Digre et al., 2017; Martin et al., 2017; O’Callaghan et al., 2018) (Table 1).

**Table 1:** Involvement of HS and heparanase expression in proteinuric diseases.

Disease/Animal Model	Species	Glomerular Heparanase Expression	HS Expression	Proteinuria	Reference
Diabetic nephropathy	Human	Increased	Reduced	(+)	Makino et al., 1992; van den Born et al., 1993; Tamsma et al., 1994; Katz et al., 2002; Maxhimer et al., 2005; van den Hoven et al., 2006; Wijnhoven et al., 2008; Garsen et al., 2016
Systemic lupus erythematosus	Human/mouse	Increased	Reduced	(+)	Seyger et al., 1998; Rops et al., 2007b; Kim et al., 2017; Szymczak et al., 2017
Membranous glomerulonephritis	Human/mouse	Increased	Reduced	(+)	van den Born et al., 1993; Garsen et al., 2016; Szymczak et al., 2017
Dense deposit disease	Human/Rat	Increased	Reduced	(+)	Smith et al., 2007; Zaferani et al., 2012
Ig A nephropathy	Human	Increased	Reduced	(+)	Sakagami et al., 2004; Celie et al., 2008; Szymczak et al., 2017
Minimal change disease	Human	Not analyzed	Reduced	(+)	Dong et al., 2009

Immortalized human glomerular endothelial cells have a glycocalyx of approximately 200 nm in culture (Singh et al., 2007). The enzymatic removal of glycans, including HS and sialic acid, increases the rate of albumin passage through the monolayers of the glomerular endothelial cells. This has been demonstrated in studies by Singh et al. (2007) and in another by Wijnhoven et al. (2007), who assessed the loss of sialic acid from the glomerular endothelial glycocalyx with neuraminidase, which was directly correlated with the increase in urinary albumin excretion. Glycan expression in the glomerular endothelium may be modulated at high glucose concentrations, which reduce the quantity of HS glycoaminoglycans and increase the passage of FITC-labeled albumin without affecting the interendothelial junctions (Singh et al., 2011). Reactive oxygen species may also cause changes to the glycocalyx of glomerular endothelial cells by depolarizing glycosaminoglycans, thereby inducing an increase in transendothelial albumin movement (Singh et al., 2013). The importance of HS in the GFB to the passage of albumin has been confirmed by assays in which heparanase knockout mice failed to develop albuminuria, whereas the albuminuria exhibited by the wild-type diabetic mice was reduced with a heparanase inhibitor (Gil et al., 2012).

## Renal Damage in Women with PE Involves a Loss of Anionic Sites in the Glomerular Endothelium

Normal pregnant women have higher levels of glomerular protein filtration than do non-pregnant women. Conversely, proteinuria above 300 mg has been detected in 24-h urine samples from women with PE (ACOG, 2013), which indicates a loss of the histological integrity of the GFB. Methods, including dextran sieving, have been described for assessing the restriction of albumin permeability. This polysaccharide is not reabsorbed or secreted by renal tubules. Therefore, it reflects the average permeability of the entire barrier. With sieving and mathematical models, researchers have identified a loss of the restriction of permeability to albumin, particularly at the end of pregnancy in women with PE, allowing for greater excretion than at the beginning of pregnancy or compared with the non-pregnant women who served as controls (Moran et al., 2003). Could the loss of anionic charges of HS from the glomerular endothelium glycocalyx explain the loss of albumin in women with PE?

This question may be partly answered with the following evidence: Histological analyses of this hypertensive disorder show that the lesions on the filtration barrier are mainly limited to endothelial cells, with no significant changes in podocytes (Lafayette et al., 1998; Strevens et al., 2003). Thus, if the damage occurs in the glomerular endothelium, some of its cellular structures must be altered (including the glycocalyx). A study has revealed a decrease in the electrical charge of the glomerular endothelium by morphometrically assessing the glomerular anionic charge and examining the pathological changes in the renal histology of African women with early onset PE (Naicker et al., 1997). Furthermore, the results showed a strong correlation between the number of anionic sites and the severity of proteinuria (Naicker et al., 1997).

Evidence supporting this earlier hypothesis and providing a more accurate approximation can be found in a more recent study. Khedun et al. (2002) evaluated 84 patients, including normotensive and proteinuric hypertensive pregnant women, as well as women with PE. This result showed a correlation between the reduction in glomerular charges and proteinuria severity. They also assessed HS levels using spectrophotometric methods with dimethylmethylene blue (DMB) and observed an increase in urinary glycosaminoglycan excretion.

The results discussed thus far indicate that HS may play a role in selective permeability as a function of the electrical charge when present in the glomerular endothelial glycocalyx. Thus, anionic charges may generate repulsion to proteins, including albumin, particularly the sulfated domains found throughout the HS chain. However, the specific chemical structures of these domains have not yet been identified.

In this scenario, it is not very clear how damage to the glomerular endothelium and the loss of HS in the glycocalyx occur. Inadequate placentation could lead to an increase in oxidative stress, thereby explaining the increased release of soluble factors including sFIt-1 (Levine et al., 2004; Goswami et al., 2006). This VEGF antagonist prevents proper communication between the podocytes that produce this antagonist and the glomerular endothelium that expresses the VEFGR receptors, thereby causing a loss of endothelial fenestrations (Sugimoto et al., 2003). Moreover, increased production of IL-1, IL-8, and TNF-α resulting from systemic inflammatory responses in women with PE could trigger glycocalyx loss due to increased heparanase activity, as will be discussed shortly (Luppi and Deloia, 2006; Bueno-Sanchez et al., 2013) (Graph 1).

## Systemic Inflammatory Response in PE Could Help to Explain the Proteinuria?

HS involvement has been described in several steps of the inflammatory process: initial binding and leukocyte rolling into the inflamed endothelium, stable adhesion of activated leukocytes to the endothelium, glycocalyx degradation, and, finally, leukocyte migration (Parish, 2006; Taylor and Gallo, 2006; Rops et al., 2007a).

Some studies have shown that HS is capable of interacting with L-selectin and *P*-selectin, thereby participating in the leukocyte rolling process of the adhesion step. Norgard-Sumnicht showed that there is an interaction between Sulfur-35-(an analog of HS with more substitutions by sulfate groups)-labeled heparin and L-selectin through a calcium-dependent process in endothelial cell cultures (Norgard-Sumnicht et al., 1993). Supporting this hypothesis, the ability of HS to bind L-selectin and *P*-selectin was assessed in *in vitro* models of endothelial cells, and a direct correlation was observed. The same study assessed the ability of heparin to interact with these adhesion molecules, and the results showed that the binding was dependent on highly sulfated NS domains, which had a higher binding affinity for adhesion molecules in heparin than HS (Norgard-Sumnicht et al., 1993).

These and other findings suggest that the HS-analog heparin could be useful as a powerful anti-inflammatory agent by inhibiting the function of L-selectin and *P*-selectin (Koenig et al., 1998). It has reported strong evidence that HS is the ligand for L-selectin in the endothelium during inflammatory processes. In this study, mice in which the enzyme *N*-deacetylase-*N*-sulphotransferase-1 (NDST-1), which is required to add sulfate groups to HS chains, was knocked out exhibited normal development, although neutrophil infiltration was observed in several tissues. These effects resulted from changes in HS that were specific to endothelial cells. In contrast, the results showed that leukocytes migrated to inflammatory sites when partially sulfated HS was expressed (Wang et al., 2005).

In addition to interacting with L-selectin and *P*-selectin, HS also interacts with cytokines to allow for leukocyte extravasation. Indeed, approximately 45 chemokines have been implicated in the recruitment of leukocytes during endothelial inflammation according to bioinformatic analyses. Furthermore, it is noteworthy that all of these chemokines have the ability to interact with HS in four possible ways at the binding sites (Lortat-Jacob et al., 2002). Reported evidence that chemokines are unable to perform their full range of functions at sites of inflammation unless they can bind to HS highlights the importance of this interaction. Different approaches have shown that HS may control the function of chemokines. First, HS may sequester chemokines by increasing the local concentration, thereby facilitating binding to its receptors that are expressed in leukocytes. Studies using IL-8 have shown that HS may induce oligomerization, and this form is more active. Similarly, the removal of glycosaminoglycan from CHO cells expressing chemokine receptors resulted in decreased binding to RANTES (regulated on activation, normal T cell expressed and secreted), MCP-1 (Monocyte Chemoattractant Protein-1), and IL-8 (Hoogewerf et al., 1997). Second, without chemokines, leukocytes are unable to form selectin-mediated stable interactions with the endothelium or migrate directionally through the endothelium. A study reported such evidence by showing that endothelium-bound chemokines induce an association between lymphocytes and their antigen receptor (lymphocyte function-associated antigen 1 – LFA1), whereas nonsoluble chemokines failed to exhibit this behavior (Shamri et al., 2005). Lastly, chemokine transport through the endothelial cell layer, known as the transcytosis process, apparently depends on HS expression (Wang et al., 2005). Concerning data from Talsma et al. (2018) indicate a pivotal role of endothelial HS in the development of renal inflammation and fibrosis in diabetic nephropathy in mice (Talsma et al., 2018). The results showed that a decrease in sulfations of endothelial HS induced an increased glomerular macrophage infiltration, mannose binding lectin complement deposition, and glomerulosclerosis (Talsma et al., 2018).

Leukocytes that bind to endothelial cells through different selectins (a process in which different cytokines are involved as mentioned above) are apparently able to cross the subendothelial space and, therefore, the basement membrane, which consists of a dense glycocalyx forming a sort of barrier. Leukocytes use several glycosidase-like enzymes that degrade glycocalyx structures to overcome the barrier. Although several enzymes have been reported, heparanase, an endo-β-glucoronidase, is the most studied in the inflammatory process. HS cleavage resulting from heparanase activity not only allows for the passage of leukocytes through the endothelium but also contributes to the increase in vascular permeability observed in inflammation (Edovitsky et al., 2006). Several studies have reported heparanase expression during the inflammatory response. Different cells, including platelets, leukocytes, neutrophils, and endothelial cells, are able to produce heparanase and degrade the extracellular matrix after stimulation with cytokines, such as IL-1, IL-8, and TNF-α (Bartlett et al., 1995; Vlodavsky et al., 1999).

Normal pregnancy is reportedly characterized by a controlled local inflammatory response, which is evident during the luteal phase of the menstrual cycle before implantation and develops as pregnancy progresses. In contrast, an exacerbated systemic inflammatory response, which may alter the endothelium and, therefore, different organs including the kidney, has been observed in PE (Sargent et al., 2007). Accumulated evidence suggests that PE might be an immune system-mediated disease, mainly an exacerbated pro-inflammatory state of pregnancy in which an injured endothelium promotes changes in leukocyte recruitment, release of pro-inflammatory cytokines, dysregulation of angiogenic pathways, and disturbances of the glomerular filtration barrier (Germain et al., 2007; Kanasaki and Kalluri, 2009). This systemic inflammation could be explained by the increase in placental factors, including soluble factors and proinflammatory cytokines, or by placental oxidative stress. The soluble receptor for VEGF, known as sFIt-1, is a possible inducer of this inflammation. sFIt-1 is able to neutralize the angiogenic functions of VEGF, and systemic inhibition would lead to generalized endothelial dysfunction because sFIt-1 is a key factor related to angiogenesis (Maynard et al., 2003). HS release from inflamed endothelium can modulate and promote some pathophysiological aspects evidenced in PE, such as the augment of neutrophil trafficking or availability of pro-inflammatory cytokines among others (Rashid et al., 2007; Goodall et al., 2014). These HS actions has not been explored in the context of inflammatory response in PE but some indirect evidences support a possible relationship between renal damage and the systemic pro-inflammatory state observed in PE.

Thus, previous reports described the histopathological finding of glomerular endotheliosis as pathognomonic of PE (Strevens et al., 2003). However, this pathological finding has been reported in women with various glomerular lesions absent from PE and in some pregnant women without complications (Strevens et al., 2003). Furthermore, an increase in proinflammatory cytokines, including TNF-α, is typical of endothelial dysfunction in pregnant women with PE (Luppi and Deloia, 2006; Rops et al., 2008; Bueno-Sanchez et al., 2013). In our laboratory, we have evaluated the effects of TNF-α, IL-1β, VEGF, and sFlt-1 in the release of heparan sulfate on a cell line of human glomerular endothelial Genc. Our fluorescence results by confocal microscopy show a decrease in the expression of 50% heparan sulfate (*P* < 0.05) after treatment with TNF-α (1, 5, and 25 ng/mL) for 24 h, largely explained by the increase in heparanase expression observed by Western blotting in a concentration dependent effect. These results were confirmed by the increase in glycosaminoglycans (800 mg/L ± 78) in culture supernatant compared to basal (400 mg/L ± 94) (Galvis-Ramirez, 2017). The enzymatic elimination of heparan sulfate induced by TNF-α contributes to the deterioration of the glycocalyx of the glomerular endothelium, which could partially explain the proteinuria observed in preeclampsia.

This inflammatory response scenario leads us to propose a hypothesis according to which PE occurs. The increase in pro-inflammatory cytokines leads to leukocyte activation and deposition in the glomerular endothelium. This triggers heparanase activity, thereby increasing glycocalyx excision, especially of HS, which would explain the loss of anionic sites in the GFB and, therefore, the associated proteinuria. Although mechanisms underlying the relation between renal disease and systemic endothelial cell dysfunction remain incompletely understood, a structural defect in the endothelial surface layer has been proposed as a mechanistic link between vascular dysfunction and albuminuric kidney disease. This approach could be common to all inflammatory diseases, as it is shown in Table 1, in which HS and heparanase are differentially expressed. Thus, inflammatory diseases whose etiology are different may promote an endothelial glycocalix dysfunction by several associated pathways and initiate albuminuria. More evidence needs to be provided in order to verify this hypothesis.

In the case of preeclampsia, this approach is based on the findings of Rops et al. (2007a), who assessed the glomerular endothelial expression of different HS domains in a mouse model of lupus nephritis and in biopsies from patients with this same condition. They observed a decrease in the *N*- and 6-*O*-sulfate domains in biopsies from patients and associated this decrease with albuminuria. A second study from the same research group assessed the adhesion of a cell line consisting of 32Dd3 granulocytes and monocytes to immortalized glomerular endothelial cells that were stimulated with two cytokines, TNF-α and IL-8. Their results showed that TNF-α activates the rolling of granulocytes and monocytes into endothelial cells, and this adhesion is mediated by HS and not by other proteoglycans. This was based on the evidence showing that the use of synthetic heparanase reduced the amount of adhesion under basal conditions, and this degraded HS was also found in the cell culture supernatant. A key observation was the increase in the relative expression of heparanase under stimuli with cytokines. However, the evaluation of the specific HS domains that may play a role in the adhesion process stands out the most among these results. Inhibition assays using antibodies directed against specific HS sequences revealed that the *N*- and 6-*O*-sulfate domains are crucial for leukocyte adhesion to the glomerular endothelium under dynamic flow conditions (Rops et al., 2008). These results and evidence reported in this review support our hypothesis, and these phenomena identified under other conditions may be occurring in the inflammatory processes that are triggered in PE.

## Conclusion

HS may play a key role in PE by interacting with different proteins through their sulfated domains. Furthermore, HS contributes to the GFB in a process known as selective permeability to the passage of albumin through the effects of the negatively charged glomerular endothelial glycocalyx. The HS-sulfated domains may contribute to this effect and, therefore, to the selective permeability of the GFB to albumin. Proinflammatory cytokines levels are increased in women with PE and systemic inflammation, which ultimately activates heparanase activity. Therefore, HS could be cleaved from the glycocalyx, which would partially explain the proteinuria observed in PE.

## Author Contributions

JQ-C, MG-R, and JB-S contributed to the conception, interpretation of data, and design of the manuscript. Additionally, all of them revised it critically for important intellectual content and provided approval for publication of the content. MG-R is a master student who looked for and selected the bibliography. JQ-C and JB-S designed the paper and proposed the hypothesis.

## Conflict of Interest Statement

The authors declare that the research was conducted in the absence of any commercial or financial relationships that could be construed as a potential conflict of interest.
